# Tissue perfusion and oxygenation to monitor fluid responsiveness in critically ill, septic patients after initial resuscitation: a prospective observational study

**DOI:** 10.1007/s10877-014-9653-8

**Published:** 2015-01-20

**Authors:** Eva Klijn, Marit H. N. van Velzen, Alexandre Pinto Lima, Jan Bakker, Jasper van Bommel, A. B. Johan Groeneveld

**Affiliations:** 1 Departments of Intensive Care, Erasmus MC University Medical Centre, PO box 2040, Room H 621, 3000 CA Rotterdam, The Netherlands; 2 Departments of Anaesthesiology, Erasmus MC University Medical Centre, Rotterdam, The Netherlands

**Keywords:** Fluid loading, Microcirculation, Passive leg raising, Microvascular oxygenation, Septic shock

## Abstract

Fluid therapy after initial resuscitation in critically ill, septic patients may lead to harmful overloading and should therefore be guided by indicators of an increase in stroke volume (SV), i.e. fluid responsiveness. Our objective was to investigate whether tissue perfusion and oxygenation are able to monitor fluid responsiveness, even after initial resuscitation. Thirty-five critically ill, septic patients underwent infusion of 250 mL of colloids, after initial fluid resuscitation. Prior to and after fluid infusion, SV, cardiac output sublingual microcirculatory perfusion (SDF: sidestream dark field imaging) and skin perfusion and oxygenation (laser Doppler flowmetry and reflectance spectroscopy) were measured. Fluid responsiveness was defined by a ≥5 or 10 % increase in SV upon fluids. In responders to fluids, SDF-derived microcirculatory and skin perfusion and oxygenation increased, but only the increase in cardiac output, mean arterial and pulse pressure, microvascular flow index and relative Hb concentration and oxygen saturation were able to monitor a SV increase. Our proof of principle study demonstrates that non-invasively assessed tissue perfusion and oxygenation is not inferior to invasive hemodynamic measurements in monitoring fluid responsiveness. However skin reflectance spectroscopy may be more helpful than sublingual SDF.

## Introduction

In critically ill, septic patients, fluid administration to improve tissue perfusion and oxygenation is generally guided by systemic haemodynamic parameters; fluid responsiveness is defined by a cardiac preload challenge by fluid infusion resulting in augmented stroke volume (SV) and cardiac output (CO) [1, 2]. Currently, several techniques are available to assess tissue perfusion, such as sidestream dark field imaging (SDF) for sublingual microcirculatory perfusion, and laser Doppler flowmetry (LDF) and reflectance spectroscopy (RS) for perfusion and oxygenation of the skin, respectively [3, 4]. Since the parameters obtained with these techniques may be sensitive predictors of outcome in critically ill and septic patients [5, 6], the effect of resuscitation measures has been studied [7, 8]. However, these studies did not focus on fluid infusion and thus do not clarify if and how tissue perfusion and oxygenation are affected by fluid responsiveness. Indeed, the effect of fluid infusion on (SDF) tissue perfusion in septic patients is controversial, regarding dependency on systemic haemodynamics, time and prior resuscitation, among others [9–12]. Indeed, in the late phase after resuscitation, the clinician may have to decide on additional fluids when the risk of harmful fluid overloading is increased. Non-invasively assessed tissue perfusion and oxygenation helping to predict and monitor fluid infusion could contribute to proper fluid management particularly at this stage.

Therefore, we studied whether tissue perfusion and oxygenation is able to predict and monitor fluid responsiveness, in critically ill, septic patients considered hypovolaemic on clinical grounds after initial resuscitation.

## Patients and methods

### Patients

This single center study was performed in the general intensive care unit (ICU) of the Erasmus MC University Medical Centre. Ethical approval for this study was provided by the ethical committee of the Erasmus MC University Medical Centre (MEC-2009-112). Written informed consent was obtained from each patient or his or her legal representative. Consecutive patients admitted in our general ICU with sepsis and on haemodynamic monitoring with a central venous catheter and a femoral artery catheter connected to a PiCCOplus™ device (Pulsion Medical Systems, Munich, Germany) were eligible. These monitoring tools are standard in our institution when sepsis is accompanied by hypotension and extensive fluid administration and vasopressor support is considered or performed. Sepsis was defined as the presence of two or more systemic inflammatory response criteria with a suspected or confirmed infection [13]. The second inclusion criterion was the presence of clinical signs of residual hypovolaemia after initial fluid resuscitation, prompting the clinical to consider additional fluid administration. These included, but were not limited to, hypotension, i.e. systolic blood pressure ≤90 mmHg, tachycardia, i.e. heart rate ≥100 bpm, central venous O_2_ saturation (S_cv_O_2_) <65 %, increasing vasopressor requirements, decreasing urine output and mottled skin. Patients were included >8 h up to 10 days after ICU admission to allow for initial resuscitation by fluid administration and vasopressor infusion and to obtain informed consent. Exclusion criteria were admission for intracranial catastrophes, known intra-abdominal hypertension, known extensive peripheral vascular disease and known congestive heart failure. Patients were treated by intensive care staff, with, among others, appropriate broad-spectrum antibiotics, source control and, if needed, intubation and mechanical ventilation according to guidelines for standard practice in our institution. None of the patients received drotrecogin alpha activated or hydrocortisone. One patient received intravenous nitroglycerin. Settings of the ventilator and of vasoactive agent infusions were unaltered during the study.

### Protocol

Patients were studied in the supine position; 250 mL of colloid solution (Voluven^®^, Fresenius Kabi, Bad Homburg, Germany) were infused in 15 min, after which measurements were repeated.

At baseline, patients were placed in supine position and after calibration and zeroing to atmospheric pressure at mid-chest level, mean arterial pressure (MAP) was taken from the femoral artery catheter and heart rate (HR) from the recorded electrocardiogram. They were measured continuously throughout the experiment. The pulse contour-derived SV and CO (PiCCOplus^®^ device, Pulsion Medical Systems, Munich, Germany) were also continuously measured. Calibration of the pulse contour-derived CO was performed by transpulmonary thermodilution involving three separate central venous injections of 20 mL of ice cold NaCl 0.9 %, at baseline. To calculate cardiac index CO was divided by body surface area. Sublingual microvascular blood flow was evaluated using SDF (MicroVision Medical, Amsterdam, The Netherlands). Image acquisition and subsequent analyses were performed according to published consensus criteria [4, 14]. In brief, after removal of saliva with gauze the device was gently applied to the sublingual area by investigators well trained in SDF imaging. For each stage five sequences of 20 s from different adjacent areas were recorded. The sequences were stored under a random number and later analysed according to the recent consensus with dedicated software (Microcirculatory Analysis Software (MAS 3.0) Academic Medical Centre, Amsterdam). Microvascular flow index was calculated after dividing each image into four equal quadrants. Quantification of flow was determined using an ordinal scale (0, no flow; 1, intermittent flow; 2, sluggish flow; 3, normal flow; 4, hyperdynamic flow) [14]. Microvascular flow index is the average score of all quadrants for a given time point. Vessel density was calculated, according to the consensus, in two manners. First, functional capillary density was calculated by measuring total length of perfused capillaries divided by image area. Second, vessel density was calculated by inserting a grid of three equidistant horizontal and three equidistant vertical lines over the image. Vessel density is equal to the number of vessels crossing these lines divided by their total length. Flow was then categorized as present, intermittent or absent, allowing calculation of the proportion of perfused vessels. In our healthy volunteers averages (median and interquartile ranges) for microvascular flow index is 3.0 [3.0–3.0] AU, for functional capillary density is 11.97 [10.5–13.1] mm/mm^2^, for vessel density is 9.9 [9.1–10.3]/mm and for proportion of perfused vessels 100 [100–100] % (unpublished data). To determine the intrarater reproducibility of the sublingual microvascular parameters, the complete image analysis on 75 randomly selected SDF sequences was repeated at a later time point, in the absence of knowledge of interventions and the intraclass correlation coefficient on consistency was calculated, considered good when ≥0.6. The intraclass correlation was 0.79 for microvascular flow index, 0.76 for functional capillary density, 0.73 for vessel density and 0.74 for proportion of perfused vessels. LDF and RS were performed using an O2C device (Oxygen to See, LEA Medizintechnik GmbH, Giessen, Germany) applied to a finger. The tissue was illuminated with a pulsed 830 nm class 1 laser diode and the backscattered light was spectrally analysed to assess the velocity-dependent frequency shifts caused by flowing red blood cells. The microvascular haemoglobin oxygen saturation and relative haemoglobin concentration were measured by illuminating tissue with visible white light (500–630 nm), which is backscattered and changed in colour according to its O_2_ saturation. The mean laser Doppler flow and microvascular haemoglobin oxygen saturation was recorded and averaged over a stable period of 1 min. In our healthy volunteers average values (median and interquartile ranges) for laser Doppler flow is 352 [185–488]AU, for microvascular haemoglobin oxygen saturation 72 [68–79] % and for relative haemoglobin concentration 47 [41–52] AU (unpublished data).

### Statistical analysis

In line with other studies and 50 % fluid responses, we estimated that 35 patients would be sufficient to reach the proof of principle study goals. Patients in whom the 250 mL fluid infusion induced an increase in SV by ≥5 % were defined as fluid responders, since the 10 % cutoff usually applies to 500 mL infusion. We nevertheless also evaluated fluid responses of ≥10 %. Non-parametric test were used because of relatively small numbers, even though most variables were distributed normally (Kolmogorov–Smirnov *P* > 0.05). Groups were compared using Mann–Whitney or Fisher exact tests, where appropriate. Intragroup comparisons were done with help of the Wilcoxon matched pairs test. Receiver operating characteristics (ROC) were calculated and compared to assess predicting and monitoring values of parameters for fluid responsiveness. Baseline values were used for prediction and the changes in parameters following the fluid challenge were used for monitoring. The areas under curves (AUC) ± standard error and sensitivity, specificity, positive and negative predictive values (PPV and NPV, respectively) are given. An AUC >0.70 was considered clinically useful. The cut-offs were determined as the cut-off values with the highest sensitivity and specificity combined. The Spearman correlation coefficient was used to express relations. The intraclass correlation coefficient was used to evaluate reproducibility of microcirculation measurements. Data are expressed as median and interquartile ranges. A two-sided *P*<0.05 was considered statistically significant. Exact *P* values are given unless <0.001.

## Results

### Baseline characteristics

The baseline characteristics of the 35 consecutive patients included in the study are shown in Table 1. The median APACHE II score and vasopressor requirements were high and almost all patients were on mechanical ventilation. Following fluid infusion, SV increased by ≥5 % in 19 (54 %) responders and by <5 % in 16 (46 %) non-responders. There were no baseline differences between the fluid response groups (Table 2).

**Table 1:** Baseline characteristics of responders and non-responders based on a ≥ 5 % increase in stroke volume to a colloid fluid infusion of 250 mL

	Responders (n = 19)	Non-responders (n = 16)	*P*
Age, year	69 [55–78]	66 [57–73]	0.46
Sex, male	11 (58)	10 (63)	0.78
Female	8 (42)	6 (37)	
BMI (kg/m^2^)	24.7 [22.5–29]	24.2 [20–27.3]	0.22
Premorbidity			
Cardiovascular	11 (58)	11 (69)	0.51
Liver disease	1 (5)	3 (19)	0.21
Malignancy	6 (32)	5 (31)	0.98
Source of sepsis			
Abdominal	7 (37)	8 (50)	0.43
Respiratory	8 (42)	5 (31)	0.51
Other	4 (21)	3 (19)	0.86
Bacteraemia	12 (63)	10 (63)	0.97
Time since admission (h)	24 [5–60]	48 [16–144]	0.16
APACHE II	28 [20–32]	24 [18–27]	0.08
SOFA	11 [7–14]	11 [8–12]	0.81
Mechanical ventilation	19 (100)	13 (81)	0.05
Vasopressor support	13 (68)	12 (75)	0.67
NE (µg kg^−1^ min^−1^)	0.06 [0–0.3]	0.18 [0–0.37]	0.55
Prior fluid balance (mL)	4,838 [2,921–10,216]	7,281 [2,725–9,537]	0.74
Survival	9 (47)	10 (63)	0.37

Median [interquartile ranges] or number and percentage, where appropriate. *BMI* body mass index, *APACHE II* acute physiology and chronic health evaluation score, *SOFA* sequential organ failure assessment, *NE* norepinephrine

**Table 2:** Haemodynamics and tissue perfusion before and after a fluid infusion, in responders and non-responders based on a ≥ 5 % increase in stroke volume to fluid infusion of 250 mL

	Responders (n = 19)			Non-responders (n = 16)		
	Before	After	*P*	Before	After	*P*
Heart rate (bpm)	89 [78–100]	92 [71–105]	0.18	96 [82–106]	95 [80–105]	0.046
Stroke volume (mL)	70 [45–87]	78 [56–97]	na	82 [65–116]	78 [64–110]	na
Cardiac output (L/min)	5.8 [5.2–7.6]*	7.0 [6.0–8.1]	<0.001	7.3 [6.6–9.9]	7.1 [6.0–8.6]	0.009
Cardiac index (L/min/m^2^)	3.3 [2.4–3.7]^†^	3.6 [3.1–4.4]	<0.001	4.0 [3.4–5.1]	3.9 [3.3–4.4]	0.009
Mean arterial pressure (mmHg)	72 [64–75]	75 [70–82]	0.001	74 [68–81]	76 [69–84]	0.16
Pulse pressure (mmHg)	64[51–75]	72 [63–85]	0.009	61 [52–82]	63 [52–84]	0.68
Microvascular flow index (AU)	3.3 [2.9–3.8]	3.9 [3.0–4.0]	0.007	3.2 [3.0–3.7]	3.6 [3.0–3.8]	0.20
Functional capillary density (mm/mm^2^)	15.0 [13.2–17.4]	16.2 [15.0–17.8]	0.030	16.0 [14.3–16.8]	15.7 [15.5–18.0]	0.24
Vessel density (mm)	10.9 [10.3–12.1]	11.9 [11.4–13.0]	0.006	11.0[10.6–13.1]	11.6 [11.2–12.7]	0.09
Proportion of perfused vessels (%)	96 [94–100]	97 [96–100]	0.06	99 [95–100]	99 [96–100]	0.14
Laser Doppler flow (AU)	115 [31–331]	169 [36–347]	0.030	270 [41–311]	287 [123–358]	0.50
µHbSO_2_ (%)	49 [26–55]	51 [43–59]	0.020	55 [38–65]	52 [40–65]	0.12
rHb (AU)	27 [23–33]	31 [26–38]	0.10	32 [25–37]	3 [25–37]	0.96

Median [Interquartile ranges]

*µHbSO*_*2*_ microvascular haemoglobin oxygen saturation, *rHb* relative haemoglobin concentration, *AU* arbitrary units, *na* not applicable

* *P* = 0.049, ^†^ *P* = 0.02 before fluid challenge in responders versus non-responders; For changes in cardiac output *P* < 0.001; mean arterial pressure *P* = 0.015; pulse pressure *P* = 0.009; rHb *P* = 0.030

### Effects of fluid infusion

Tables 3 and 4 show the haemodynamic and microcirculatory values for fluid responders and non-responders, when defined on the basis of ≥5 and 10 % increase in SV, respectively. The SV, CO, MAP and pulse pressure (PP) increased in responders but not in non-responders. In responders (and not in non-responders), microvascular flow index, vessel density, functional capillary density, laser Doppler flow and microvascular haemoglobin concentration and oxygen saturation increased.

**Table 3:** Haemodynamics and tissue perfusion before and after a fluid infusion, in responders and non-responders based on an ≥10 % increase in stroke volume to fluid infusion of 250 mL

	Responders (n = 15)			Non-responders (n = 20)		
	Before	After	*P*	Before	After	*P*
Heart rate (bpm)	89 [78–120]	89 [71–118]	0.12	98 [82–101]	97 [80–104]	0.09
Stroke volume (mL)	59 [43–75]*	70 [55–97]	na	82 [68–116]	82 [66–116]	na
Cardiac output (L/min)	5.5 [4.7–6.6]^†^	6.7 [5.9–8.0]	0.001	7.5 [7.0–9.9]	7.3 [6.5–8.6]	0.35
Cardiac index (L/min/m^2^)	2.9 [2.2–3.6]^‡^	3.4 [3.0–4.1]	0.001	4.0 [3.4–5.2]	3.9 [3.4–4.8]	0.082
Mean arterial pressure (mmHg)	72 [64–75]	77 [71–82]	0.002	73 [68–81]	75 [69–84]	0.040
Pulse pressure (mmHg)	64 [46–74]	71 [63–79]	0.021	63 [56–82]	64 [55–86]	0.36
Microvascular flow index (AU)	3.2 [2.9–3.8]	3.9 [3.0–4.0]	0.006	3.2 [3.0–3.8]	3.5 [3.0–3.8]	0.24
Functional capillary density (mm/mm^2^)	15.2 [13.2–17.4]	16.3 [15.0–17.8]	0.046	15.5 [14.2–17.2]	15.9 [15.3–18.0]	0.14
Vessel density (mm)	11.1 [10.3–12.1]	12.1 [11.4–13.0]	0.019	10.9 [10.5–12.8]	11.7 [10.9–12.8]	0.032
Proportion of perfused vessels (%)	97 [94–100]	98 [97–100]	0.13	98 [93–100]	99 [95–100]	0.041
Laser Doppler flow (AU)	216 [31–347]	278 [36–365]	0.035	203 [41–303]	221 [51–313]	0.49
µHbSO_2_ (%)	51 [34–59]	54 [47–61]	0.006	52 [20–60]	51 [22–64]	0.27
rHb (AU)	27 [21–29]	31 [26–38]	0.030	32 [25–37]	33 [25–37]	0.60

Median [interquartile ranges]

*µHbSO*_*2*_ microvascular haemoglobin oxygen saturation, *rHb* relative haemoglobin concentration, *AU* arbitrary unit, *na* not applicable

* *P* = 0.005, ^†^ *P* = 0.002, ^‡^ *P* = 0.001 responders versus non-responders before fluid challenge; for changes in cardiac output *P* < 0.001; mean arterial pressure *P* = 0.011; pulse pressure *P* = 0.006; µHbSO2 *P* = 0.024; rHb *P* = 0.042

**Table 4:** Monitoring values of haemodynamic parameters for fluid responsiveness

	AUC ± Std. error *P*		Optimal cutoff sens		Spec	PPV	NPV
SV ≥ 5 %							
Delta PP (mmHg)	0.76 ± 0.09	0.003	4	68	88	87	70
Delta MAP (mmHg)	0.74 ± 0.09	0.006	6	58	88	85	64
Delta rHb (AU)	0.72 ± 0.09	0.015	−0.7	44	93	68	73
SV ≥ 10 %							
Delta PP (mmHg)	0.77 ± 0.09	0.004	6	73	90	85	82
Delta MAP (mmHg)	0.75 ± 0.09	0.006	6	73	90	85	82
Delta µHbSO_2_ (%)	0.73 ± 0.09	0.015	2	64	79	69	75
Delta rHb (AU)	0.71 ± 0.09	0.025	1.5	50	89	78	71
Delta MFI (AU)	0.69 ± 0.10	0.048	0	71	69	67	73

*AUC* area under the curve, *Std*. standard, Sens sensitivity, Spec specificity, *PPV* positive predictive value, *NPV* negative predictive value, *PP* pulse pressure, *SV* stroke volume, *MAP* mean arterial pressure, *μHbSO*_*2*_ microvascular haemoglobin oxygen saturation, *rHb* relative haemoglobin concentration, *MFI* microvascular flow index

### Prediction and monitoring of fluid responsiveness

Except for baseline SV and CO there were no baseline predictors among global haemodynamic and tissue perfusion variables. Table 4 shows the ROC curves for monitoring of fluid responsiveness ≥5 and 10 %, by changes in variables (except for SV and CO). The AUC values indicative of global hemodynamics and tissue perfusion did not differ from each other.

### Correlations

The increase in SV related to the increase in rHb (r_s_ = 0.41, *P* = 0.014) and µHbSO_2_ (r_s_ = 0.38, *P* = 0.086).

## Discussion

Our results suggest that in critically ill, septic patients with clinical hypovolemia after initial resuscitation and persistent fluid responsiveness, fluid infusion augments several indicators of tissue perfusion, so that these non-invasively derived indicators can be used to monitor fluid infusion.

In fact, our results suggest vascular recruitment and flow increments (SDF) by fluid infusion in responders. However, the effect of fluid infusion and the subsequent increase in SV on the parameters of tissue perfusion was relatively small in our study, and changes in SDF variables in responders did not differ from that in non-responders. We included patients beyond the initial phase of fluid resuscitation and SDF measurements suggest that the sublingual microcirculation was sometimes hyperdynamic at this stage. De Backer et al. [12] noted amelioration of microcirculatory alterations in the course of sepsis. This was also seen in the study by Boerma et al. [8] who observed similar values for sublingual microvascular perfusion as in our study, at 24 h after admission and administration of approximately 6 L of fluids. The study by Pottecher et al. [11] demonstrated a much larger effect of fluids on SDF measurements. A possible explanation for the discrepancy with our study could be that their patients were likely to be more severely hypovolaemic than ours. Indeed, their patients were included early during resuscitation, i.e. within 24 h after admission, although the fluid balance before inclusion is unclear. Additionally, baseline microcirculatory perfusion was lower than in our study. Conversely, ‘late’ inclusion may mitigate an effect of fluid administration on tissue perfusion (SDF) [10], but in our study effects seemed independent of time from admission.

Tissue perfusion parameters seemed, at least in part, dependent on systemic haemodynamics. This is in agreement with some studies [11] but in contrast to other observations, early (<24 h) and late (>48 h) in the disease course of critically ill, septic patients [9, 10, 12]. However, prior resuscitation had not similarly affected perfusion and oxygenation of sublingual and cutaneous tissue. In contrast to SDF, LDF and RS parameters were still in the low range (compared to healthy volunteers) at baseline in our study [5]. A poor skin and sublingual perfusion is often observed in septic patients, and has been identified as a sensitive predictor of outcome, independent from systemic haemodynamic parameters [5, 6]. The redistribution of CO with regional over- and underperfusion relative to demand is a central hemodynamic abnormality of septic shock [14]. The fact that both systemic and sublingual perfusion were sometimes judged hyperdynamic could point to a close relationship between the two. However, relatively low skin perfusion and oxygenation was associated with a relatively low SV and CO, in patients responding to fluid infusion. This suggests that peripheral (rather than sublingual) tissue blood flow is partly dependent on total forward flow. Conversely, fluid administration had a greater effect on LDF/RS- than on SDF-derived parameters. Of the tissue perfusion parameters studied, changes in skin haemoglobin concentration and saturation were most helpful in monitoring fluid responsiveness.

The limitations of our study include the relatively small number of patients. Future research on guiding fluid resuscitation by tissue perfusion parameters is also needed to study benefits of this approach of increasing tissue oxgenation by fluid administration in critically ill, septic patients, since our study suggests reasonable intrarater and intrapatient reproducibility of some of these non-invasive measurements. Additionally, we cannot exclude that prediction and monitoring values of microcirculatory parameters are different from ours in patients with less prior resuscitation. Finally, real time analysis would be needed to utilise SDF images for guiding fluid administration, the necessary off-line analysis makes it currently unsuitable for clinical use.

In conclusion, the value of non-invasively assessed skin perfusion and oxygenation for monitoring fluid responsiveness in critically ill, septic patients after initial resuscitation is not inferior to that of invasive hemodynamic measurements. This may help fluid management in septic patients, even though an outcome benefit has yet to be demonstrated.
